# Altered social attention in anorexia nervosa during real social interaction

**DOI:** 10.1038/srep23311

**Published:** 2016-03-17

**Authors:** Mario Dalmaso, Luigi Castelli, Pietro Scatturin, Lorenza Carli, Patrizia Todisco, Daniela Palomba, Giovanni Galfano

**Affiliations:** 1 Dipartimento di Psicologia dello Sviluppo e della Socializzazione, Università di Padova, Italy; 2 Centro di Neuroscienze Cognitive, Università di Padova, Italy; 3 Centro per i Disturbi del Comportamento Alimentare, Casa di Cura Villa Margherita, Arcugnano, Italy; 4 Dipartimento di Psicologia Generale, Università di Padova, Italy

## Abstract

The capacity to devote attentional resources in response to body-related signals provided by others is still largely unexplored in individuals with Anorexia Nervosa (AN). Here, we tested this capacity through a novel paradigm that mimics a social interaction with a real partner. Healthy individuals (Experiment 1) and individuals with AN (Experiment 2) completed a task with another person which consisted in performing, alternatively, rapid aiming movements to lateralised targets. Generally, this task leads to a form of Inhibition of Return (IOR), which consists of longer reaction times when an individual has to respond to a location previously searched by either himself (individual IOR) or by the partner (social IOR) as compared to previously unexplored locations. IOR is considered as an important attentional mechanism that promotes an effective exploration of the environment during social interaction. Here, healthy individuals displayed both individual and social IOR that were both reliable and of the same magnitude. Individuals with AN displayed a non-significant individual IOR but a reliable social IOR that was also significantly stronger than individual IOR. These results suggest the presence of a reduced sensitivity in processing body-related stimuli conveyed by oneself in individuals with AN which is reflected in action-based attentional processes.

The term Anorexia Nervosa (AN) refers to a severe psychiatric disease in which individuals show a drastic reduction in food intake and are obsessed by having a thin body shape. People who suffer from AN are mainly women^1^ and the mortality rate in this clinical population is one of the highest among other psychiatric diseases^2^. AN represents also a great challenge to the scientific community as little is known about its causes^3^ and efforts are made in order to develop increasingly refined diagnosis and therapies^4^.

Besides the aberrant eating behaviours that severely impact on the physical health of individuals with AN, evidence is accumulating reporting alterations also in the domain of many cognitive processes related to socio-emotional processes^5^. For instance, individuals with AN exhibit impairments both in the processing of social rewards^6^ and in emotional functioning^7^,^8^. In addition, they seem to be less sensitive to social signals provided by others, such as emotional expressions^9^,^10^, and they tend to avoid direct eye contact^11^. In this regard, the capacity to devote attentional resources in response to spatial cues provided by others, such as eye gaze direction, but also pointing gestures, head and body turns, is universally considered as a key feature of human behaviour necessary to successfully navigate within social contexts^12^. This kind of ‘social attention’ allows individuals to discover objects or entities of potential interest in the environment, as well as to program and coordinate social behaviour in interpersonal contexts^13^.

Recent evidence has been provided suggesting that individuals with AN show an altered attentional processing in response to different spatial cues (including eye gaze), in the presence of a spared ability to shift attention following cues related to body parts (a schematic arm with a finger pointing either leftwards or rightwards^14^). This latter pattern has been interpreted as evidence for a bias for processing body parts of others that finds a growing support in the literature^15^,^16^,^17^,^18^. For instance, a recent eye-tracking study showed that individuals with AN spend much more time exploring body parts of others as compared to both face and eyes, when inspecting visual images^6^. Furthermore, individuals with AN appear to show a better visual discrimination of body parts of others as compared to healthy controls^19^.

Experimental research in the domain of social attention in individuals with AN has so far mainly relied on well-established cognitive paradigms that share a common feature, namely the fact that the participants take part in tasks that are not intrinsically social^15^. Indeed, the tasks basically require to participate individually by providing a response to stimuli presented on a computer screen. These paradigms allow for a close control of independent variables but sometimes they do not fully reveal how we use social attention in everyday life when other individuals are present^13^. Recently, Welsh and his collaborators^20^ have proposed a novel and clever paradigm that mimics real social interactions without sacrificing methodological robustness. In this task, two participants (e.g., Participants A and B) sit across from each other and are asked to perform rapid aiming movements to targets that randomly appear rightwards or leftwards on a response device placed between them (see Fig. 1, Panel A). Crucially, responses are provided alternatively, so that Participant A provides two single responses, followed by two single responses from Participant B, and then the sequence is repeated starting with new responses from Participant A. In so doing, two measures can be obtained: one relative to the influence of the previous action made by each participant on her/his own upcoming action (within-person trials); another one relative to the influence of the action of one participant on the upcoming action of the other (between-person trials). In healthy individuals, this type of task elicits higher Response Times (RTs) for targets appearing in the same spatial location as in the previous trial as compared to targets appearing in a different location^20^, a well-known phenomenon called Inhibition of Return (IOR) that is thought to reflect a hard-wired bias towards scanning unexplored locations preventing spatial attention to return on recently inspected portions of space^21^,^22^. The novelty of the task proposed by Welsh and collaborators^20^ was that a reliable IOR emerged both on within-person trials (individual IOR) and on between-person trials (social IOR^23^), and these two effects were also of the same magnitude. Social IOR has been mainly interpreted as the consequence of the fact that the basic mechanism underlying IOR is also responsible of keeping track of actions and orienting of attention performed by other individuals^13^,^20^.

A version of this paradigm has recently been implemented to investigate social attention in individuals with Autism Spectrum Disorder (ASD)^24^. Individuals with ASD performed the task with a healthy confederate, and exhibited a spared individual IOR in the face of a reduced social IOR. These results confirmed the presence of a reduced sensitivity to spatial signals provided by others in individuals with ASD. Importantly, this evidence was achieved by asking participants to interact with a real person instead of responding to stimuli delivered on a computer screen, an approach that allowed to tremendously increase the ecological validity of the results^13^.

In the present study, we designed two experiments with the aim of exploring social attention in individuals with AN during real social interactions by using the paradigm proposed by Welsh and collaborators^20^. The goal of Experiment 1 was to test the experimental apparatus with healthy individuals and to replicate the original findings reported by Welsh and collegues^20^. The goal of Experiment 2 was to address individual and social IOR in individuals with AN. In light of recent evidence suggesting a high sensitivity in processing body-related stimuli conveyed by others in individuals with AN^6^,^15^,^16^,^17^,^18^,^19^, we expected these individuals to exhibit a robust IOR for responses provided by others (i.e., a stronger social rather than individual IOR).

## Experiment 1: Healthy individuals

### Method

#### Participants

Twenty healthy students (*Mean age* = 22.8 years, *SD* = 1.11, *Mean years of education* = 16.8, *SD* = 1.11, 16 females, all right-handed) at the University of Padova took part in this study on a voluntary basis. All participants were naïve to the purpose of the experiment. The study was conducted in accordance with the guidelines laid down in the Declaration of Helsinki and was approved by the Ethics Committee for Psychological Research of the University of Padova. Informed consent was obtained from all participants.

#### Materials and procedures

**Figure 1:**
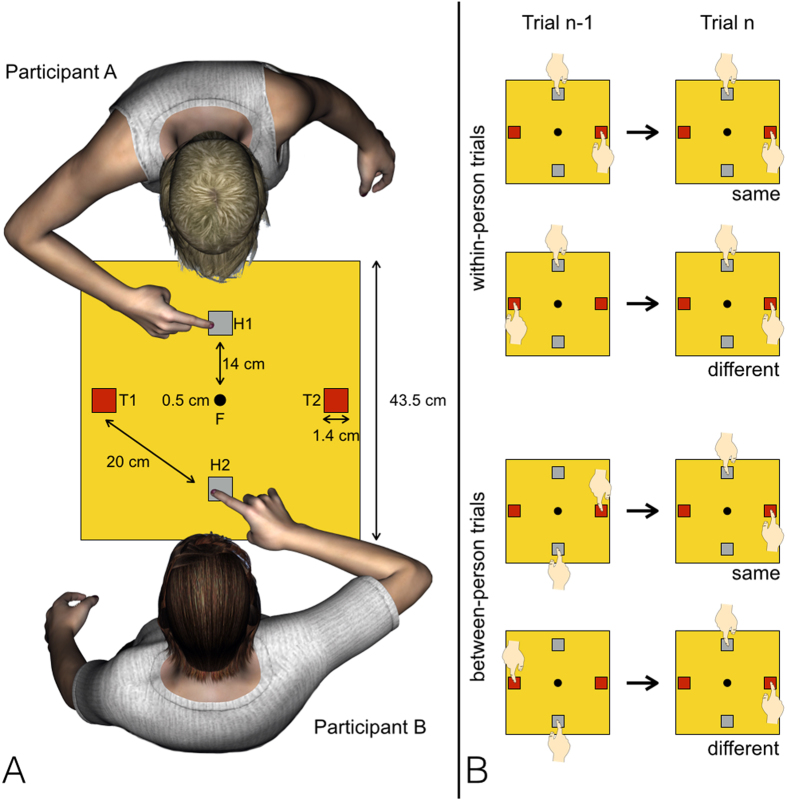
Illustration of the apparatus and the experimental conditions. Panel **A** shows a top-view illustration (not drawn to scale) of the experimental apparatus and its size, and of the placement of the two participants (**A** and **B**). H1 and H2 represent the “home” buttons while T1 and T2 represent the “target” buttons. The central dot represents the fixation point on which participants maintained their gaze for the whole duration of each block. Panel **B** shows a schematic illustration of within- and between-person trials divided by target location.

Materials and procedures were similar to those used by Welsh and collegues^20^. Participants were divided in same-gender couples. Members of each couple (hereafter, participant A and participant B, see Fig. 1, panel A) sat in front of each other. In between them there was the experimental apparatus. This was composed of a metallic board connected to a PC running a custom program created with E-Basic for E-Prime 1.1. Four buttons were located on the surface of the board. A “home” button was located in front of each participant (H1 and H2, see Fig. 1, panel A). The remaining two buttons, which contained a red Light Emitting Diode (LED), represented the “target” buttons and were located one on the right and one on the left of participants (T1 and T2, see Fig. 1, panel A).

Participants were asked to keep their eyes fixed on a central point (a central dot, see Fig. 1, panel A) and were instructed to keep their “home” buttons pressed, with the index finger of their dominant hand. Subsequently, one of the two “target” buttons randomly flashed for 100 ms, and a specific participant, for instance A, was asked to depress the “home” button as fast as possible and perform a rapid aiming movement in order to press the “target” button that had just been flashed. After that, 1000 ms were allowed to return to the “home” button and press it. The “target” button that was flashed in trial *n* had the same probability to be the same as that in trial *n-1* (i.e., same target; see Fig. 1, panel B), or different with respect to that in trial *n-1* (i.e., different target location). The comparison of performance in same and different target location conditions enabled an estimation of IOR.

The experiment proceeded with a planned sequence of responses. More specifically, the participants were instructed to respond to two consecutive trials, so that two trials were responded to by participant A, the next two trials were responded to by participant B, in an AABBAABBAA…(etc.) pattern. This design allowed for separate estimations of IOR as a function of whether the respondent on trial *n* was the same (AA, BB) or different (AB, BA) with respect to the one actively involved on trial *n-1.* This, in turn, enabled us to examine both within-person (i.e., individual) and between-person (i.e., social) IOR effects (see Fig. 1, panel B).

Overall, each couple of participants responded to 660 experimental trials. As proposed by Welsh and colleagues^20^, Response Time (RT) was measured from the onset of the LED contained in the “target” button to the release of the “home” button and represented the key dependent variable. Movement Time (MT, measured from the release of the “home” button to the pressure of the “target” button) was also recorded and analyzed, although it is thought to reflect more peripheral processing and hence is less informative for investigating social attention and cognitive processing stages^23^. In so doing, RTs and MTs were two numerically independent measures. There were 4 practice blocks, each composed of 16 trials, followed by 20 experimental blocks, each composed of 33 trials in which both “target” buttons were flashed for an equal number of times. Because the very first trial could not be informative with regards to IOR, the first respondent provided an additional response at the end of each block. This ensured to have the same number of trials in each cell of the experimental design.

### Results

#### Data pre-processing

The very first trial of each block was eliminated from the analyses because, for its nature, it was not preceded by a response. Similarly, trials in which participants broke the proper alternation pattern (2.4% of total trials) were excluded. No localization errors were made. Median RTs and MTs were submitted to two different repeated-measure ANOVAs with target location (same vs. different) and person who provided the response in the previous trial (same vs. different) as within-participant factors. The Bayesian Information Criterion (BIC) was also computed in order to test the robustness of the results^25^,^26^. Indeed, this criterion is particularly useful for dealing with the null hypothesis appropriately, as it disentangles whether the null or the alternative hypothesis is more corroborated by the data. In the present context, BIC values higher than 0.50 indicate that there is more evidence for the alternative than for the null hypothesis, whereas values lower than 0.50 indicate the opposite.

#### Data analysis

With regards to RTs, the main effect of target location was significant, *F*(1, 19) = 60.547, *p* < 0.001, *η*^*2*^_*p*_ = 0.761, 95% CI [0.502, 0.849], owing to shorter RTs in response to different-location trials (*M* = 279 ms, *SE* = 7.3, 95% CI [264,295]) than to same-location trials (*M* = 300 ms, *SE* = 8.25, 95% CI [283,317]), as well as the main effect of person, *F*(1, 19) = 9.585, *p* = 0.006, *η*^*2*^_*p*_ = 0.335, 95% CI [0.035, 0.568], owing to shorter RTs in response to same-person trials (*M* = 283 ms, *SE* = 6.99, 95% CI [269,298]) than to different-person trials (*M* = 296 ms, *SE* = 8.83, 95% CI [278,315]). Importantly, the target location × person interaction was not significant (*F* < 1, *p* = 0.641). Indeed, two-tailed paired t-tests confirmed the presence of both within-person IOR, *t*(19) = 5.782, *p* < 0.001, *d*_*z*_ = 1.293, 95% CI [12.65, 27.00], *p*_BIC_(H1 | D) > 0.99, and between-person IOR, *t*(19) = 6.209, *p* < 0.001, *d*_*z*_ = 1.388, 95% CI [14.53, 29.32], *p*_BIC_(H1 | D) > 0.99.

With regards to MTs, the main effect of target location was not significant (*F* = 2.409, *p* = 0.137), whereas the main effect of person was significant, *F*(1, 19) = 7.368, *p* = 0.014, *η*^*2*^_*p*_ = 0.279, 95% CI [0.012, 0.526], owing to shorter MTs on same-person trials (*M* = 245 ms, *SE* = 10, 95% CI [226,264]) than on different-person trials (*M* = 251 ms, *SE* = 10.39, 95% CI [229,273]). The target location × person interaction approached significance, *F*(1, 19) = 3.573, *p* = 0.074, *η*^*2*^_*p*_ = 0.158, 95% CI [ < 0.001, 0.422]. Paired two-tailed t-tests revealed that MTs were not different for between-person trials, *t*(19) = 0.603, *p* = 0.554, *d*_*z*_ = 0.135, 95% CI [−2.35, 4.25], whereas for within-person trials there was a trend towards shorter MTs in response to same location than different location trials, *t*(19) = −2.006, *p* = 0.059, *d*_*z*_ = −0.449, 95% CI [−10.94, 0.23], a result in line with that reported by Welsh and colleagues^20^.

Overall, these results confirmed the presence in healthy participants of both individual and social IOR, both reliable and of same magnitude, replicating previous findings^20^.

## Experiment 2: Individuals with anorexia nervosa

### Method

#### Participants

Twenty individuals with AN (*Mean age* = 25.5 years, *SD* = 10.15, *Mean years of education* = 11.45, *SD* = 3.22, all females and right-handed; restrictive subtype, *n* = 10; binge-purge subtype, *n* = 10) were recruited from a private clinic located in northern Italy. Diagnoses of AN were made by a board-certified attending research team of senior psychiatrists through the Structured Clinical Interview^27^ of the Diagnostic and Statistical Manual of Mental Disorders (DSM-IV). Exclusion criteria were board-certified diagnosis of cognitive or personality disorders, psychosis, mental retardation and major depression. Seventeen individuals were medicated. Most common medications were neuroleptics and/or SSRI antidepressants. Vitamins, diet supplements, gastrointestinal medications, or benzodiazepines at need were also included. All participants were naïve to the purpose of the experiment. The study was conducted in accordance with the guidelines laid down in the Declaration of Helsinki and was approved by the Ethics Committee for Psychological Research at the University of Padova. Informed consent was obtained from all participants.

#### Clinical measures

Several clinical measures were available about individuals with AN at the time of testing and consisted of self-reported tests (see Table 1). The Beck Depression Inventory^28^ (BDI-II) was used to assess the severity of depression; the Bulimic Investigatory Test Edinburgh^29^ (BITE) was used to assess bulimic behaviours; the Clinical Impairment Assessment^30^ (CIA) was used to assess psychosocial impairments due to eating disorders; the Eating Attitudes Test^31^ (EAT-40) and the Eating Disorder Examination Questionnaire^32^ (EDE-Q) were used to assess eating disorders; the Symptom Checklist-90-Revised^33^ (SCL-90-R) was used to assess a broad range of psychological problems and symptoms related to psychopathology.

#### Materials and procedures

Materials and procedures were the same as that used in Experiment 1, with only two exceptions: firstly, following the same approach used by Welsh and collegues^24^, individuals with AN completed the task with a confederate of similar demographic characteristics (24 years, female, right-handed) recruited from a non-clinical population in order to mimic everyday interactions effectively. Secondly, the length of the experiment was slightly reduced in order to avoid fatigue in individuals with AN. There were 4 practice blocks, each composed of 14 trials, followed by 14 experimental blocks, each composed of 33 trials. In so doing, each couple responded to 462 experimental trials.

### Results

#### Data pre-processing

Data of individuals with AN were analyzed in the same manner as in Experiment 1, namely, we eliminated the very first trial of each block as well as trials on which participants broke the proper alternation pattern (5% of total trials). No localization errors were made. Median RTs and MTs were submitted to two distinct repeated-measure ANOVAs with target location (same vs. different) and person (same vs. different) as within-participant factors. BIC scores were also computed, as in Experiment 1.

#### Data analysis

With regards to RTs, the main effect of target location was significant, *F*(1, 19) = 27.380, *p* < 0.001, *η*^*2*^_*p*_ = 0.590, 95% CI [0.248, 0.741], owing to shorter RTs in response to different-location trials (*M* = 387 ms, *SE* = 17.68, 95% CI [350,424]) than to same-location trials (*M* = 416 ms, *SE* = 20.03, 95% CI [374,458]), as well as the main effect of person, *F*(1, 19) = 8.770, *p* = 0.008, *η*^*2*^_*p*_ = 0.316, 95% CI [0.026, 0.553], owing to shorter RTs in response to same-person trials (*M* = 387 ms, *SE* = 20.01, 95% CI [345,428]) than to different-person trials (*M* = 417 ms, *SE* = 18.7, 95% CI [378,456]). More importantly, the target location × person interaction was also significant, *F*(1, 19) = 14.365, *p* = 0.001, *η*^*2*^_*p*_ = 0.431, 95% CI [0.092, 0.636]. Two-tailed paired t-tests revealed the presence of a reliable between-person IOR, *t*(19) = 7.696, *p* < 0.001, *d*_*z*_ = 1.721, 95% CI [32.62, 56.98], *p*_BIC_(H1 | D) > 0.99, and a non-significant within-person IOR, *t*(19) = 1.695, *p* = 0.106, *d*_*z*_ = .379, 95% CI [−3.14, 29.89], *p*_BIC_(H1 | D) = 0.48 (see Table 2).

With regards to MTs, the main effect of target location was not significant (*F* < 1, *p* = 0.864), whereas the main effect of person was significant, *F*(1, 19) = 11.314, *p* = 0.003, *η*^*2*^_*p*_ = 0.373, 95% CI [0.055, 0.596], owing to shorter MTs on same-person trials (*M* = 450 ms, *SE* = 48.43, 95% CI [349,552]) than on different-person trials (*M* = 547 ms, *SE* = 60.24, 95% CI [421,673]). The target location × person interaction was not significant (*F* < 1, *p* = 0.371). Indeed, two-tailed paired t-tests confirmed that MTs did not differ as function of target location on both within- and between-person trials (*t*s ≤ 0.765, *p*s ≥ 0.453).

In order to control for any effect due to subtypes of AN, if any, we conducted two further ANOVAs for RTs and MTs with target location (same vs. different) and person as within-participant factors, and with subtype of AN (restrictive subtype vs. binge-purge subtype) as between-participant factor. Results remained virtually unchanged. As concerns RTs, the main effects of target location and person, as well as the target location × person interaction, were significant (*F*s > 8.316, *p*s < 0.01), while subtype of AN did not lead to any significant main or interaction effect (*F*s < 1.136, *p*s > 0.301). As concerns MTs, the only significant result remained the main effect of person, *F*(1, 18) = 10.762, *p* = 0.004, *η*^*2*^_*p*_ = 0.374, 95% CI [0.048, 0.588], while the subtype of AN did not lead to any significant main or interaction effect (*F*s < 1.221, *p*s > 0.284).

Finally, correlation techniques were also employed to explore any potential relationships between IOR and demographic/clinical measures of individuals with AN. Firstly, we calculated an index of the magnitude of IOR (i.e., RT on same target location trials – RT on different target location trials) divided by person for both social and individual components. Then, these indices were correlated with BMI index, age of illness onset, duration of illness, and the other clinical test measures reported in Table 1. No significant results emerged (*p*s > 0.187; “false discovery rate” correction). Importantly, the lack of a correlation between IOR and BDI-II scores excluded that depression – a clinical variable that often shows comorbidity with AN – played a relevant role in shaping the attentional effects reported here. Overall, we can conclude that the main results observed in individuals with AN confirmed the presence of a very peculiar pattern of social attention characterized by a reduced individual IOR in the face of a strong social IOR.

#### Comparison between Experiments 1 and 2

In order to provide a more direct evidence that the performance of individuals with AN differed from that of healthy individuals, we conducted an additional exploratory analysis on RTs by comparing two roughly matched subgroups extracted from the two experiments. These two subgroups were obtained by removing male participants from Experiment 1 (*n* = 4) and then by removing participants whose age fell three times over the interquartile range of the two merged groups, as assessed by a boxplot analysis. This latter procedure identified four extreme values, all belonging to the sample tested in Experiment 2. In so doing, we obtained two groups with the same numerosity (*n* = 16), matched for gender (all females), handedness (all right-handed) and age, *t*(30) = 1.208, *p* = 0.237, *d*_z_ = 0.441. Education was higher in the control group, *t*(30) = 6.651, *p* < 0.001, *d*_z_ = 2.429. However, it is highly unlikely that education played any critical role in this type of paradigm assessing basic cognitive processes.

After that, we conducted a mixed-design ANOVA on RTs with target location (same vs. different) and person (same vs. different) as within-participant factors, and group (healthy individuals vs. individuals with AN) as between-participant factor. The results confirmed that the two groups performed the task differently. Indeed, the three-way target location × person × group interaction was significant, *F*(1, 30) = 6.729, *p* = 0.015, *η*^*2*^_*p*_ = 0.183, 95% CI [0.007, 0.402]. Two further ANOVAs were subsequently performed separately for the two groups, since a within-participants design provides better control of individual differences. As for healthy individuals, the main effect of target position was significant, *F*(1, 15) = 44.334, *p* < 0.001, *η*^*2*^_*p*_ = 0.747, 95% CI [0.425, 0.846], and it was no further qualified by the interaction with person (*F* < 1, *p* = 0.556). Indeed, both the individual and the social IOR were present (*t*s ≥ 4.75, *p*s ≤ 0.001, *p*sBIC(H1 | D) > 0.99). As for individuals with AN, the main effect of target position was significant, *F*(1, 15) = 19.209, *p* < 0.001, *η*^*2*^_*p*_ = 0.562, 95% CI [0.166, 0.734], as well as the target position × person interaction, *F*(1, 15) = 11.532, *p* = 0.005, *η*^*2*^_*p*_ = 0.435, 95% CI [0.061, 0.653], due to a non-significant individual IOR (*p* = 0.151, *p*BIC(H1 | D) = 0.44) in the face of a robust social IOR (*p* < 0.001, *p*BIC(H1 | D) > 0.99). Finally, as suggested by one reviewer, the three-way target location × person × group interaction was also splitted by person rather than by group. In line with our main hypothesis, the two-way target location × group interaction was significant for social IOR (*F* = 8.442, *p* = 0.007) but not for individual IOR (*F* < 1, *p* = 0.624).

Overall, we can conclude that the main results confirmed the presence of a very specific pattern of social attention in individuals with AN.

## Discussion

The study of social-cognitive abilities in AN is still largely unexplored. However, evidence is accumulating showing that individuals with AN display alterations in some domains of social cognition^6^,^7^,^8^ and, more specifically, they exhibit abnormal attentional responses to signals provided by other individuals, mostly when body parts are concerned^11^,^14^. Almost invariably, previous studies have capitalized upon well-established paradigms derived from experimental psychology that, however, often present oversimplified social settings in which the perception and reaction to stimuli presented on computer screens is assessed^13^.

The aim of the present study was to investigate the capacity to shift attention in response to spatial cues provided by others in individuals with AN in a more ecological setting. To this purpose, we adopted a recent paradigm^20^ apt to assess social attention during real social interactions with another individual.

In healthy individuals, this task generally elicits both individual and social IOR, both reliable and of the same magnitude^20^, and several recent studies have deeply explored this phenomenon^13^,^34^,^35^. Data from Experiment 1, in which healthy individuals were tested, confirmed this pattern of results. Indeed, healthy participants were slower to initiate an aiming movement towards a spatial location that had just been explored either by themselves (individual IOR) or by the partner (social IOR) as compared to a novel spatial location. Importantly, these two effects were both significant and of comparable magnitude. This pattern confirms that the adopted paradigm was effective in measuring the two types of IOR.

In Experiment 2, the same task was performed by a sample of individuals with AN. The results showed a clear asymmetric response to oneself and the other. Indeed, individuals with AN displayed a reliable and strong social IOR, indicating that they were affected by the previous behavioural response of the interaction partner. In contrast, they exhibited a non-significant individual IOR which, in turn, suggests an alteration in spatial attention due to an ineffective integration of previous exploratory behaviour performed by themselves. Even if the lack of significance for individual IOR was unexpected, it should be noted that, according to some recent evidence, individuals with AN seem to display a reduced sensitivity in the processing of information conveyed by their own body. For instance, a recent neuroimaging study has reported that, when presented with images of their own body, individuals suffering from eating disorders exhibit a decreased activation of cortical brain areas strongly associated to attention processing such as the inferior parietal lobule^18^. Vocks and collaborators^18^ concluded that this finding is likely to reflect a bias against processing one’s own body in individuals with eating disorders. Furthermore, another neuroimaging study reported that when individuals with AN were presented with images of their own body, no significant activations emerged as compared to a baseline, whereas significant activations were reported in response to others’ body^36^. Finally, a recent behavioural study documented that individuals with AN would have more difficulties in recognizing their own face as compared to faces of others^37^. In the present context, such one’s own body-related avoidance effect would be reflected in a reduced capacity to take into account and integrate one’s own behavioural response in the planning and execution of following actions. Moreover, this pattern could be also interpreted according to data indicating a bias towards processing body parts of others^14^,^15^,^16^,^17^,^18^,^19^, as confirmed even in eye-tracking studies^6^.

A crucial question emerging from the present results is whether individuals with AN could display a non-significant individual IOR even when they are tested in isolation – for instance, by employing a classic cue-target Posner-like paradigm^21^ which does not require any reaching movement. This approach could unveil the presence of an impairment involving very basic mechanisms underlying IOR not necessarily related to social attention and body representations. Furthermore, another important question that arises from the present results is whether the use of a confederate only in Experiment 2 could have played a potential role in shaping our results. However, we can reasonably rule out this possibility. Indeed, in an independent study – with a different purpose to that reported in the present paper – we had 13 healthy female students completing the same task as that reported in Experiment 1 together with a healthy female confederate. In other words, as in Experiment 2, the confederate was kept constant for all the participants. Similar to Experiment 1, the main effect of target location was significant, *F*(1,12) = 16.320, *p* < 0.001, *η*^*2*^_*p*_ = 0.576, 95% CI [0.132, 0.752], whereas the interaction between target location and person was not significant (*F* < 1, *p* = 0.459). Indeed, both individual and social IOR were reliable (*t*s ≥ 2.84, *p*s ≤ 0.02, *p*sBIC(H1 | D) ≥ 0.89).

The present findings are theoretically consistent with several recent experimental evidence^38^,^39^ showing that individuals with AN display disturbances in the domain of body schema, which consists of a mental representation of the body and encodes information on body size and shape as well as information about the exact location of the body and its parts in environmental space^40^. One possibility is that individuals with AN in the present study exhibited a null individual IOR as a result of their difficulties in updating a proper mental representation of their body that takes into account the executed exploratory behaviours of their hand in the environment. This possibility is so far speculative, but it would be important to explore this scenario in the future by combining the present IOR paradigm with more classical measures aimed at assessing the body schema. Nonetheless, the present findings are consistent with data from healthy participants that suggest an involvement of distinct brain areas when processing one’s own and others’ body parts^41^.

Individual and social IOR have been recently investigated also in another specific clinical population, namely individuals with ASD^24^. Because social cognition is a domain that appears to be severely compromised both in individuals with AN and in individuals with ASD^42^, this line of investigation based on the administration of similar paradigms to assess different disorders may be extremely helpful to determine the extent to which the same or different cognitive processes are affected in the two populations. In this regard, Welsh and colleagues^24^ have reported a deficit in the social cognition domain in individuals with ASD in the form of a reliable individual IOR in the face of a reduced social IOR. In contrast, the present findings with individuals with AN show a reduced individual IOR in the face of a reliable social IOR. Overall, it seems that while the notion of an alteration in social cognition processes can be confirmed in both individuals with ASD and AN, however such alteration emerges in very different ways.

To conclude, the present study represents the first attempt to investigate social attention in AN during real social interactions. The results observed in individuals with AN speak in favour of a hyposensitivity to their own actions in the face of a remarkable attentional response towards actions executed by others.

Results from the present study can be also considered in light of models of attention bias for disorder-salient stimuli in patients with eating disorders^43^,^44^. Recently, psychological treatments for eating disorders have been proposed aimed at diverting patients’ attention from anxiety-provoking thoughts or visual stimuli. Attentional bias modification treatments (ABMT) have been successfully applied with EDs patients as a way to implicitly retune automatic attentional bias^45^. Evidence from the present study could constitute a first step towards structuring and implementing treatment plans which include ABMT as a targeted intervention for those individuals with AN who exhibit significant attentional bias towards behaviours displayed by others in social interaction contexts.

## Additional Information

**How to cite this article**: Dalmaso, M. *et al.* Altered social attention in anorexia nervosa during real social interaction. *Sci. Rep.* **6**, 23311; doi: 10.1038/srep23311 (2016).

## Figures and Tables

**Table 1:** Clinical information of individuals with AN.

Variable		Mean score (SD)
Body Mass Index (BMI)		15.9 (2.23)
Age of illness onset (years)		16.9 (4.93)
Duration of illness (years)		8.9 (9.23)
Back Depression Inventory (BDI-II)	*Global score*	33.1 (9.65)
	*Item 9 (suicide symptoms)*	1.2 (.89)
Bulimic Investigatory Test Edinburgh (BITE)	*Symptoms*	14.1 (6.57)
	*Gravity*	6.6 (6.52)
Clinical Impairment Assessment (CIA)		31 (9.26)
Eating Attitudes Test (EAT-40)		58.4 (21.89)
Eating Disorder Examination Questionnaire (EDE-Q)	*Restraint*	18.4 (9.39)
	*Eating concern*	16.2 (8.73)
	*Shape concern*	34.7 (12.79)
	*Weight concern*	19.7 (8.89)
The Symptom Checklist-90-Revised (SCL-90-R)		179.25 (48.59)

*Note*. Higher scores for clinical tests indicate higher levels of impairment. BMI (individual scores): 14.65, 12.50, 17.91, 19.77, 15.21, 16.02, 19.69, 15.04, 15.35, 16.1, 15.26, 13.25, 16.61, 19.03, 14.95, 14.36, 16.83, 11.56, 18.29, 15.58.

**Table 2:** Median RTs and MTs for each condition presented in Experiments 1 and 2.

	Same person		Different person	
	Same target location	Different target location	Same target location	Different target location
*Exp.1: Healthy participants*				
RTs	293 (7.82)	273 (6.51)	307 (9.47)	285 (8.52)
MTs	242 (9.03)	247 (9.36)	252 (10.38)	251 (10.46)
*Exp.2: Individuals with AN*				
RTs	393 (22.65)	380 (17.85)	439 (19.18)	394 (18.67)
MTs	459 (52.96)	442 (50.42)	534 (58.48)	560 (66.43)

*Note.* Values in brackets are SEM.
